# Tooth Graft and Platelet‐Rich Fibrin Mixture for Oral Bone Reconstruction and Preservation: A Scoping Review

**DOI:** 10.1002/cre2.70160

**Published:** 2025-07-31

**Authors:** Mina Shekarian, Fateme Aghajani, Zahra Sadat Mohajer Hejazi, Pedram Iranmanesh, Bahamin Mousa Akbari‐Bezenjani, Abbasali Khademi

**Affiliations:** ^1^ Research Assistant, Dental Research Center, Dental Research Institute, School of Dentistry Isfahan University of Medical Sciences Isfahan Iran; ^2^ School of Dentistry, Student Research Committee Azad University, Isfahan (Khorasgan) Branch Isfahan Iran; ^3^ Department of Endodontics, Dental Research Center, Dental Research Institute, School of Dentistry Isfahan University of Medical Sciences Isfahan Iran

**Keywords:** bone regeneration, platelet‐rich fibrin, PRF, socket preservation, tooth graft

## Abstract

**Aim:**

This scoping review aimed to evaluate effectiveness of using tooth graft (TG) mixture with platelet‐rich fibrin (PRF), examining their roles in oral bone reconstruction and preservation, and evaluating the clinical and histopathological outcomes associated with this approach.

**Material and Methods:**

Articles were searched in Scopus, Medline, Web of Science, ProQuest, and Google Scholar up to November 2024. The search was guided by the PCC framework: Population: alveolar bone and calvaria bone defects; Concept: the use of a TG mixture with PRF as a bone graft material; Context: clinical and histopathological outcomes of oral bone reconstruction and preservation. Two independent researchers carried out the screening, study selection, and data extraction processes.

**Results:**

Use of the PRF and TG mixture for socket preservation is superior to using each of these biomaterials individually. However, in the context of bone reconstruction, although all studies confirm new bone formation, the superiority of this mixture over the separate use of each biomaterial remains uncertain. Additionally, all studies confirm the increase in bone density and new bone formation aimed at implant placement.

**Conclusion:**

The majority of studies have reported that the PRF and TG mixture enhances both the quality and quantity of bone.

## 1 Introduction

Oral bone reconstruction and preservation are critical treatments in dentistry, especially in the context of tooth extraction, trauma, periodontal disease, or congenital defects. The long‐term success of dental implants and overall oral rehabilitation significantly depends on effective bone regeneration strategies (Tumedei et al. 2019). Traditional bone grafting procedures often rely on autografts, allografts, or xenografts; however, limitations such as donor site morbidity, immune reactions, infection risk, and cost have prompted the search for safer, biologically active, and cost‐effective alternative (Gual‐Vaqués et al. 2017). In recent years, the search for biomaterials that not only promote healing but also mimic the natural properties of bone and overcome all these limitations has intensified.

Recent advancements in regenerative dentistry have led to increased interest in autologous materials that exhibit both osteoconductive and osteoinductive properties while eliminating the risks associated with foreign graft materials. Two such materials—tooth‐derived grafts (TG) and platelet‐rich fibrin (PRF)—have individually shown promising outcomes in bone healing applications. More recently, the combined use of TG and PRF has been proposed as a novel strategy to enhance bone regeneration, capitalizing on their complementary biological effects (Adamska et al. 2024; Singh et al. 2023; Gowda et al. 2023; Alrmali et al. 2023).

Extracted teeth, once considered medical waste, are now being repurposed as valuable autogenous grafting materials due to their compositional similarity to alveolar bone, including hydroxyapatite content, collagen, and bone morphogenetic proteins (BMPs) (Inchingolo et al. 2023; Minetti et al. 2023; Picone et al. 2024). Using a patient's own teeth as a bone graft material (autogenous tooth bone graft) is a feasible and effective technique for regenerating bone in the jaw. This approach offers several advantages, including a high degree of bone regeneration and a reduced risk of rejection or infection compared to other types of bone grafts (Gual‐Vaqués et al. 2017).

Likewise, PRF, a second‐generation platelet concentrate, is rich in growth factors that support angiogenesis and bone remodeling, making it a widely used material in socket preservation and implant‐related procedures (Afifi et al. 2024; Jia et al. 2024; Strauss et al. 2018). While each component alone has demonstrated significant regenerative potential, their synergistic use is hypothesized to result in superior outcomes. The PRF matrix may enhance the handling and stability of TG particles while sustaining the release of growth factors that accelerate healing. Conversely, the structural matrix provided by the TG may support the organized infiltration of regenerative cells facilitated by PRF. Research indicates that both PRF and TG independently exhibit strong bone regeneration properties (Gowda et al. 2023; Mohammed 2021; Ouyyamwongs et al. 2019). However, no comprehensive review exists on the efficacy of their combined use for oral bone reconstruction and preservation.

This scoping review aimed to provide an overview of the available literature on the use of a TG mixture with PRF, examining their roles in oral bone reconstruction and preservation, and evaluating the clinical and histopathological outcomes associated with this approach. By assessing current research findings, this review helps clarify the benefits and limitations of this biomaterial combination in the context of dental and maxillofacial bone reconstruction.

## 2 Materials and Methods

### 2.1 Protocol and Registration

The study protocol was developed based on the framework by Peters et al. (2015), as outlined by the Joanna Briggs Institute, and was registered at Open Science with 10.17605/OSF. IO/PMQV4 (https://osf.io/yc9nb/). This scoping review was conducted following the PRISMA (Preferred Reporting Items for Systematic Reviews and Meta‐analyses) extension for scoping reviews (Tricco et al. 2018) and reported in accordance with the PRISMA‐Scr Checklist, as outlined in Table S2.

### 2.2 Objective

The objective of this study was to assess the efficacy of a TG and PRF mixture as a bone graft material for augmenting oral bone defects.

### 2.3 Eligibility Criteria

The randomized and non‐randomized controlled clinical trials (RCTs), cohort studies, case reports, and case series that evaluated the use of TG and PRF for bone reconstruction or preservation were included. Exclusion criteria encompassed animal and in vitro studies, electronic posters, letters to the editor, review articles, and book chapters. In addition, to enhance the overall quality and minimize bias, studies with a high risk of bias were excluded (Mohammed 2021; Kim et al. 2021; Melek and El Said 2017).

### 2.4 Information Source and Search Strategy

An electronic search was conducted without time restrictions in following databases: Scopus, Medline, Cochrane, Web of Science up to November 2024, limited to English‐language. (((((((((((Autologous bone graft) OR (ATG)) OR (Tooth graft)) OR (dentin)) OR (human dentin)) OR (DDM)) OR (dentin matrix)) OR (dentin graft)) OR (ATB)) OR (Teeth derived graft)) AND ((PRF) OR (Platelet rich fibrin)))) in TITLE/SUBJECT/ABSTRACT (Table S1) was used for the electronic search. Additionally, ProQuest (T&D), 100 first hit of Google Scholar, reference lists of included studies searched. The review followed the Population‐Concept‐Context (PCC) framework. The Population included cases with alveolar and calvaria bone defects. The Concept focused on the use of a TG combined with PRF as a bone graft material. The Context encompassed the evaluation of clinical and histopathological outcomes related to oral bone reconstruction and preservation.

### 2.5 Screening Process

After the removal of duplicates using EndNote reference manager software (version 21) and manual review, two independent reviewers (Z.M. and F.A.) screened the titles and abstracts. The full text of the records was screened using the eligibility criteria. Disagreements between reviewers were resolved through discussion, and if necessary, a third reviewer (M.S.) was consulted. If full‐text access was restricted, the corresponding authors were contacted.

### 2.6 Data Extraction

Key data were extracted from each included study, summarized in Table 1. The data included the first author's last name, year of publication, study design, sample size, age range, study duration and follow‐up, TG and PRF provided protocol, risk of bias, and reported outcomes, including vertical and horizontal bone reconstruction, new bone formation, alveolar ridge width, crestal height, and other relevant measures. The Delphi technique was conducted to reach a consensus on the extracted data (Nasa et al. 2021). Any disagreement was resolved through discussion with other reviewers (M.S.). If data were missing, the co‐author was contacted via email.

**Table 1:** Detailed characteristics of included articles.

Study design	References	Graft technique	Number of cases	Age range	Study period	TG and PRF provided protocol	Reported outcomes	Risk of bias
***Socket preservation***								
RCT	Amer et al. (2024)	Test group: TG + i‐PRF Control group: TG	12	Test group (mean): 36.5 ± 10.8 Control group (mean): 34.5 ± 4.9	6 months	Dentin particle size: NM Demineralization agent: HCL i‐PRF: 700 rpm during 3 min TG/i‐PRF: 1/1	Ridge width Ridge height Keratinized tissue width Postoperative pain	Low
	Gowda et al. (2023)	Test group: TG + A‐PRF or i‐PRF Control group: A‐PRF+ plug alone	16	20–55	4 months	Dentin particle size: 300–1200 μm Demineralization agent: EDTA A‐PRF: 1300 rpm during 8 min i‐PRF: 700 rpm during 3 min TG/PRF:1/1	Crestal height Ridge width Ridge height	Moderate
	Sah and Baliga (2022)	Test group: TG + PRF Control group: PRF membrane	20	NM	NM	Dentin particle size: NM Demineralization agent: NM PRF: NM TG/PRF: NM	BD Trabecular score Bone healing Pain Lamina dura score	Moderate
	Ouyyamwongs et al. (2019)	Test group: TG + PRF Control group: PRF alone	40	NM	8 weeks	Dentin particle size: 500–700 μm Demineralization agent: NM PRF: 3000 rpm during 10 min	Ridge dimension Ridge width Ridge height Bone healing	Moderate
Case series	van Orten et al. (2022)	TG + i‐PRF	7	28–73	26 months	Dentin particle size: 300–1200 μm Demineralization agent: EDTA i‐PRF: 700 rpm during 3 min for women, and 4 min for men	Histological evaluation Cancellous new bone formation	Low
	Pohl et al. (2020)	TG + PRF	13	Over 18	Approximately 4 months	Dentin particle size: 300–1200 Demineralization agent: EDTA PRF: 1300 rpm during 8 min TG/PRF: 2/1	Dimensional ridge changes BBT, BPR, RW, LH Histological examination	Low
	Andrade et al. (2020)	TG + L‐PRF	10	Older than 18 years	6 months	Dentin particle size: 300–1200 Demineralization agent: EDTA L‐PRF: 2800 rpm/480 g/12 min TG/L‐PRF: 1/1	Vertical and horizontal dimensions of the alveolar ridge Histological evaluation	Low
Case report	Adamska et al. (2024)	TG + A‐PRF	1	A 21‐year‐old patient	12‐month follow‐up	Dentin particle size: 300–1200 Demineralization agent: EDTA A‐PRF: 1500 rpm during 14 min	Bone defect reconstruction	Low
***Bone Reconstruction***								
Cyst treatment								
Case report	Vares et al. (2022)	TG + PRF	1	NM	NM	Dentin particle size: 300–1200 Demineralization agent: EDTA A‐PRF: 1300 rpm during 8 min TG/PRF: N.M	Healing Steointegration	Low
***Implant placement***								
RCT	Abdelraheim et al. (2023)	Test group: TG + PRF around Control group: TG	6	20–35	6 months	Dentin particle size: 300–1200 μm Demineralization agent: EDTA PRF: 3000 rpm during 10 min TG/PRF: NM	Horizontal and vertical bone loss BD Pain, swelling or infection Implant stability	Moderate
	ElAmrousy and Issa (2022)	Test group: TG + L‐PRF Control group: TG	26	NM	N.M	Dentin particle size: 300–1200 μm/Demineralization agent: NaOH L‐PRF: 2800 rpm/400 g/12 min	New bone formation, Reduced marginal bone loss, Mesiodistal bone gain	Moderate
Case report	Singh et al. (2023)	TG + i‐PRF	1	35	4 months	Dentin particle size: 500–1200 μm Demineralization agent: HNO_3_ i‐PRF: 700 rpm during 3 min	Quality and quantity bone formation Implant support	Low
	Khunger (2021)	PRF + TG	1	25	3 months	Dentin particle size: NM Demineralization agent: HNO_3_ PRF: NM	Alveolar bone resorption	Low
Case series	Alrmali et al. (2023)	TG + PRF – 4 socket preservation cases, – 5 cases of guided tissue regeneration, – 5 cases of GBR, – 10 cases of sinus augmentation procedures, – 2 immediate placement implants – 2 socket shields.	26	Over 18 years	follow‐up of 32 months	Dentin particle size: 300–1200 μm/Demineralization agent: EDTA PRF: 2700 rpm/400 g during 12 min	Bone coverage over implant platforms Histological analysis: newly formed bone.	Low

Abbreviations: A‐PRF = advanced‐platelet‐rich fibrin, BBT = buccal bone plate thickness, BD = bone density, BIC = bone‐to‐implant contact, BMP‐2 = bone morphogenetic protein‐2, BPR = buccal bone plate reduction, CAL = clinical attachment level (mm), CD = crevicular depth, GBR = guided bone regeneration, GR = gingival recession, HCAL = horizontal clinical attachment level, i – PRF = injectable platelet‐rich fibrin, LH = lingual ridge height, L‐PRF = leukocyte and platelet‐rich fibrin, MBL = marginal bone level (mm), NM = not mentioned, OFD = open flap debridement, PDDM = partial dentin demineralized matrix, PD = probing depth, PRF = platelet‐rich fibrin, RW = ridge width, RH = ridge height, rhBMP‐2 = recombinant human bone morphogenetic protein‐2, TG = tooth graft, VBL = vertical bone level, VCAL = vertical clinical attachment level, WAG = width of attached gingiva (mm).

### 2.7 Critical Appraisal Within Sources of Evidence

The Joanna Briggs Institute (JBI) critical appraisal tools were utilized to evaluate the quality of case series, case reports, and RCTs (Munn et al. 2020; Moola et al. 2017; Barker et al. 2023). The Delphi technique was conducted to reach a consensus (Nasa et al. 2021).

Moderate risk of bias studies were included in this review because the topic remains novel, and the body of existing literature is still limited. Excluding them would have resulted in an incomplete representation of the current state of research. To address this, we clearly reported the risk of bias in Tables S3–S5, allowing readers to interpret the findings in the context of study quality. Moreover, we avoided drawing strong conclusions based on studies with high bias.

### 2.8 Outcome Measures

The primary outcomes assessed were vertical and horizontal bone reconstruction, new bone formation, alveolar ridge width, and crestal height. Secondary outcomes included other reported parameters such as clinical attachment level, ridge resorption, and additional bone density or volume measurements.

## 3 Results

### 3.1 Selection of Sources of Evidence

**Figure 1:**
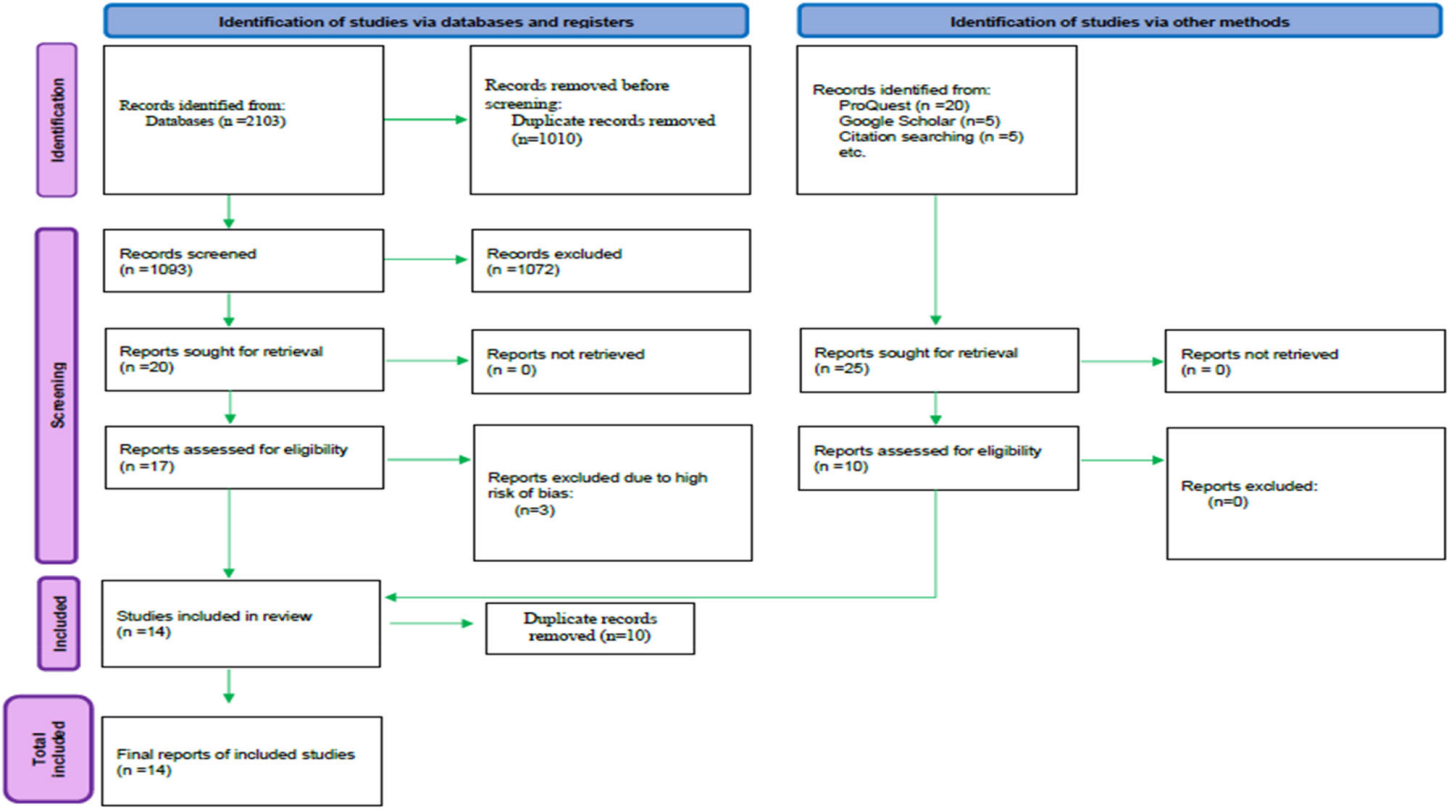
PRISMA flowchart for selecting articles.

The initial search resulted in 447 records from Medline, 565 from Scopus, 447 from Web of Science, 644 from Embase. After removing duplicates (1010 records), 1096 records were excluded during the initial screening of titles and abstracts. Three records were excluded at the full‐text evaluation phase due to the high risk of bias (Mohammed 2021; Kim et al. 2021; Melek and El Said 2017). No additional records were included from gray literature sources, as the studies collected from gray literature were duplicates of those obtained from the databases and were therefore eliminated. Ultimately, 14 records published between 2017 and 2024 were included (Figure 1).

### 3.2 Critical Appraisal Within Sources of Evidence

**Figure 2:**
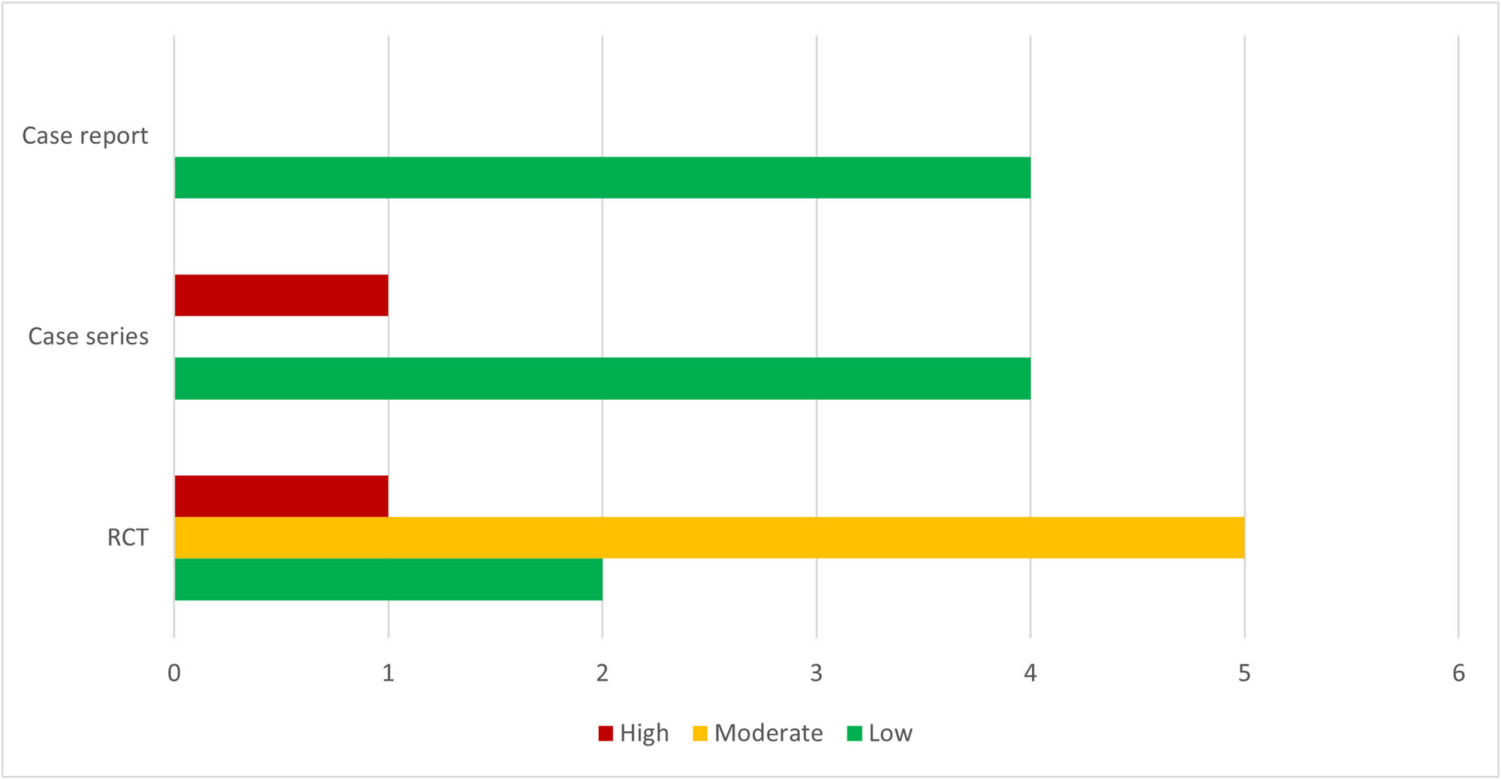
Assessment of risk of bias level in each type of study.

Among 14 included studies, 9 had low, 5 had moderate bias, which is shown separately in Figure 2 for each type of study. The Cohen's kappa coefficient was assessed at 0.91 for inter‐examiner agreement. Details of risk of bias are represented in Tables S3–S5.

### 3.3 Characteristics of Records

Table 1 summarize the main data. According to the study purpose, eight records evaluated socket preservation, one study represented bone reconstruction, and rest of them were about implant placement.

### 3.4 Socket Preservation

Research investigating the use of TG combined with PRF for socket preservation includes two case reports (Adamska et al. 2024), three case series (Andrade et al. 2020; Pohl et al. 2020; van Orten et al. 2022), and three RCTs (Gowda et al. 2023; Ouyyamwongs et al. 2019; Sah and Baliga 2022). These studies present conflicting findings regarding vertical and horizontal bone changes.

### 3.5 Vertical Bone Height

Gowda et al. (2023). reported a significant increase in the socket's vertical dimension, as well as buccal and palatal/lingual cortical height, when partially demineralized TG was combined with A‐PRF or i‐PRF, compared to A‐PRF alone. Similarly, Andrade et al. (2020) noted that the vertical dimension of the alveolar ridge was either preserved or increased in patients treated with TG and L‐PRF. Adamska et al. (2024) also used a mixture of A‐PRF and tooth graft during socket preservation and tooth transplantation, which successfully preserved alveolar bone and promoted bone regeneration in the defect area. Pohl et al. (2020) confirmed an increase in bone height when TG is combined with PRF. Amer et al. (2024) observed that the ridge height showed less reduction in the i‐PRF and TG group compared using TG alone. Conversely, Ouyyamwongs et al. (2019) observed that the difference in vertical resorption was not significant in PRF alone compared to the PRF combined with the TG group.

### 3.6 Horizontal Ridge Width

Gowda et al. (2023) found improvements in horizontal crestal width in groups treated with TG and PRF. Conversely, Ouyyamwongs et al. (2019) observed that PRF alone led to significantly greater horizontal ridge width compared to the PRF combined with the TG group. Pohl et al. (2020), however, reported a decrease in horizontal ridge width and buccal bone plate thickness following treatment with PRF and TG, while Andrade et al. (2020) observed maintenance or a slight increase in ridge width. Amer et al. (2024) further reported in their study that the group using the i‐PRF and TG mixture experienced greater width reduction compared to the group using TG alone.

### 3.7 Bone Density and Healing Quality

Sah and Baliga (2022) reported that combining PRF with TG resulted in accelerated bone healing, increased bone density, and improved trabecular scores at a 6‐month follow‐up, compared to PRF alone. Similarly, Ouyyamwongs et al. (2019) noted that faster bone healing was observed when TG was added to PRF.

### 3.8 Histological Findings

Although most studies reported clinical and radiographic parameters, Andrade et al. (2020) and Pohl et al. (2020) included histological evaluations that confirmed new bone formation and favorable structural remodeling.

### 3.9 Bone Reconstruction

Among the studies evaluating the combination of PRF and TG for bone reconstruction, a case report (Vares et al. 2022) was included. Vares et al. (2022) reported that the application of A‐PRF combined with TG was highly effective in regenerating bone within a relatively large defect in a short period.

### 3.10 Implant Placement

Since the quality and quantity of bone at the implant site are critical for successful treatment outcomes, several studies have explored the effectiveness of using a mixture of PRF and TG as a bone graft biomaterial. Among the studies aimed at implant placement, two were RCTs, two were case reports, and two were case series.

### 3.11 Ridge Dimensions at Implant Site

Several studies have assessed the influence of combining PRF with TG on ridge preservation at implant sites. Abdelraheim et al. (2023) demonstrated that this combination effectively reduced both horizontal and vertical bone loss compared to TG alone. On the other hand, ElAmrousy and Issa (2022) represented that adding the L‐PRF (leukocyte‐ and platelet‐rich fibrin) to the TG did not cause to make a significant difference between horizontal bone alteration, while marginal bone loss was significantly diminished and bone gain was significantly increased. van Orten et al. (2022) reported preserved ridge dimensions with cancellous bone formation, allowing for later implant placement without the need for further interventions.

### 3.12 Implant Stability

Abdelraheim et al. (2023) also reported a marked improvement in implant stability in the group treated with the PRF and TG mixture compared to the group that received TG alone. These findings suggest that the biologically active components of PRF may enhance early osseointegration and contribute to long‐term implant success.

### 3.13 Histological Evaluations

van Orten et al. (2022) confirmed that dentin granules used in the graft were surrounded by newly formed bone, with ongoing remodeling observed up to 4 months after grafting. These findings further support the biological compatibility and regenerative potential of the PRF‐TG mixture in implant‐related applications.

## 4 Discussion

The regenerative outcomes of combining TG and PRF appear to be influenced by some critical variables, including the size of dentin particles and the type and preparation of PRF used.

PRF has various types, including L‐PRF, A‐PRF (advanced platelet‐rich fibrin), i‐PRF, and A‐PRF+ , which are categorized as second‐generation platelet‐rich derivatives, each with its own mechanical, structural, and biological properties that influence their regenerative performance (Simoes‐Pedro et al. 2022). The key differences lie in the centrifugation speed and duration, which influence the concentration of growth factors, cells, and the quality of the fibrin (Eren et al. 2016; Dohan Ehrenfest et al. 2018).

The TG preparation involves several steps to demineralize the dentin while preserving its bioactive components. The protocols may vary based on the company that you are using their devices or clinical application, but they generally include the following stages: Tooth selection and cleaning, pulpectomy and fragmentation, demineralization, sterilization, and particle size adjustment (Minetti et al. 2022). The protocols and materials used for preparing TG vary significantly among companies, especially during the demineralization stage. Some protocols use HCl, others rely on EDTA, HNO_3,_ and some employ alternative demineralizing agents (Minetti et al. 2022). Additionally, differences are observed in the particle sizes of dentin used in these protocols. For instance, certain protocols utilize dentin particles with sizes ranging from 300 to 1200 micrometers, while others opt for smaller particle sizes between 500 and 700 micrometers (Ouyyamwongs et al. 2019; Minetti et al. 2022). These variations in the preparation methods of demineralized dentin matrix can significantly influence its bone regeneration potential. The choice of demineralizing agent affects the retention of bioactive components, such as growth factors and proteins, while the particle size impacts the scaffold's ability to support cellular attachment, migration, and bone formation.

### 4.1 Socket Preservation

The TG–PRF mixture generally demonstrated better outcomes than PRF or TG alone in terms of vertical ridge height and buccal/palatal cortical bone preservation (Gowda et al. 2023; Ouyyamwongs et al. 2019; Pohl et al. 2020; Sah and Baliga 2022; Amer et al. 2024). Despite the general agreement among most studies regarding the significant effectiveness of this mixture in preserving the vertical dimension of bone, its effectiveness in maintaining the horizontal dimension of the bone remains unclear. Some studies have reported it as minimal (Pohl et al. 2020), others as less effective than PRF alone (Ouyyamwongs et al. 2019), while some have found it to be significantly effective (Gowda et al. 2023). Additionally, the study by Ouyyamwongs et al. (2019) is the only one to reported did not observe a significant difference in vertical bone resorption comparing PRF alone with PRF mixed TG.

These discrepancies may stem from variations in the protocols used for preparing TG and PRF across studies. Gowda et al. (2023) employed A‐PRF and i‐PRF, prepared at 3000 rpm for 10 min, while Pohl et al. (2020) utilized PRF prepared at 1300 rpm for 8 min. Differences in centrifugation speed and duration can significantly impact the concentration of cells and growth factors, the structural integrity of PRF, its longevity, and ultimately its regenerative potential. Additionally, differences in dentin particle size and the TG preparation protocol may contribute to these discrepancies in results. While Pohl et al. (2020) and Gowda et al. (2023) used particles ranging from 300 to 1200 micrometers and employed the KomitoBio protocol for TG preparation, Ouyyamwongs et al. (2019) utilized particles sized between 500 and 700 micrometers and followed the protocol provided by SPEXSamplePrep. The larger particle size (300–1200 micrometers) may offer better interaction with surrounding tissues, provide structural support, and cause less immune system stimulation, which can enhance the regenerative potential compared to smaller particles.

### 4.2 Bone Reconstruction

Significant bone gain and improved clinical outcomes when L‐PRF is used as a sole grafting material or in combination with other biomaterials have been highlighted in previous studies (Otero et al. 2022). Although all studies have confirmed the effectiveness of the TG and PRF mixture in promoting bone formation, there is no consensus on whether this mixture is superior to using PRF or TG alone. While some studies have claimed that using this mixture significantly enhances bone formation and the density of the newly formed bone, others have refuted this assertion. The discrepancy may be attributed to differences in the protocols used for preparing PRF and TG.

### 4.3 Implant Placement

Studies investigating bone formation aimed at implant placement have shown that a mixture of PRF and TG has the ability to promote new bone formation, with a significant increase in bone density compared to the use of TG alone (Singh et al. 2023; Abdelraheim et al. 2023; ElAmrousy and Issa 2022; Khunger 2021). Additionally, implant stability in the group where PRF was added to TG was significantly higher in long‐term follow‐ups compared to the group without PRF (Abdelraheim et al. 2023). Moreover, beyond its use in combination with TG, the application of liquid PRF alone as a coating for implant surfaces has also shown promising outcomes (de Oliveira Fernandes et al. 2022). Therefore, it appears that the use of the PRF and TG mixture for implant placement can be effective.

Overall, the variations in study outcomes may be attributed to differences in PRF preparation protocols, such as the speed and duration of centrifugation, the type of PRF used, and variations in TG preparation protocols, including the size of dentin particles, the type of demineralization agent applied, or the ratio of TG to PRF in the mixture. Additionally, differences in the surgical site and study design could also contribute to these discrepancies in results.

### 4.4 Strength, Limitations, Gap, and Future Suggestion

To the best of our knowledge, this is the first scoping review to evaluate the efficacy of combining PRF and TG as a biomaterial. Due to the novelty of this topic, relatively few RCTs have been conducted. The existing studies exhibit low homogeneity, and most have a moderate risk of bias, making statistical analysis infeasible.

While the majority of studies suggest that combining PRF and its derivatives with TG improves both the quantity and quality of bone, the null hypothesis cannot be definitively rejected. In addition, previous studies did not specify the type of newly formed bone, whether it was lamellar or woven. This omission limits the interpretation of the regenerative outcomes. The significant heterogeneity among the studies likely contributes to this outcome. Given the critical role of increasing bone quantity and quality in improving dental treatments, such as implant procedures, further well‐designed RCTs are strongly recommended in this field.

## 5 Conclusion

Although the majority of studies have reported that the PRF and TG mixture enhances both the quality and quantity of bone, the cost‐effectiveness of using this mixture compared to TG or PRF alone remains unclear and requires further investigation.

## Author Contributions

**Mina Shekarian:** conceptualization, data gathering, data Analysis, writing – original draft, writing – review and editing. **Fatemeh Aghajani:** data gathering, writing – original draft. **Zahra Sadat Mohajer Hejazi:** data gathering, writing – original draft. **Pedram Iranmanesh:** writing – review and editing. **Bahamin Mousa akbari‐Bezenjani:** data analysis, writing – original draft, writing – original draft. **Abbasali Khademi:** conceptualization, data gathering, data analysis, writing – original draft, writing – review and editing. The manuscript is an original work of the author. All data, tables, figures, etc., used in the manuscript were prepared originally by the authors.

## Ethics Statement

Since this is a review article that does not include human or animal subjects, ethics approval was not required. However, the study protocol was registered on the Open Science platform with the ID number 10.17605/OSF.IO/PMQV4.10.17605/OSF. IO/PMQV4. We confirm that our submitted manuscript has been prepared in our personal capacity and not as official representatives or on behalf of the Iran government entity. Our work is conducted independently, with no affiliations or dependencies on any government.

## Conflicts of Interest

The authors declare no conflicts of interest.

## Supporting information

Table S1.

Table S2.

Table S3.

Table S4.

Table S5.

## Data Availability

The data that support the findings of this study are available from the corresponding author upon reasonable request.
