# Supplementary material for: Tooth Graft and Platelet‐Rich Fibrin Mixture for Oral Bone Reconstruction and Preservation: A Scoping Review
Source: Clin Exp Dent Res. 2025 Jul 31;11(4):e70160. doi: 10.1002/cre2.70160 (PMC12311611; doi:10.1002/cre2.70160)
Supplement: Supplementary file 1 — Table S1. [file CRE2-11-e70160-s004.docx]

| Data Base | Search Strategy |
| --- | --- |
| WOS | TS= (“Autologous bone graft” OR “ATG” OR “Tooth graft” OR “dentin” OR “human dentin” OR “DDM” OR “dentin matrix” OR “dentin graft” OR “ATB” OR “Teeth derived graft”) AND (“PRF” OR “Platelet rich fibrin”) |
| Medline | ("Autologous bone graft"[Title/Abstract] OR "ATG"[Title/Abstract] OR "Tooth graft"[Title/Abstract] OR "dentin"[Title/Abstract] OR "human dentin"[Title/Abstract] OR "DDM"[Title/Abstract] OR "dentin matrix"[Title/Abstract] OR "dentin graft"[Title/Abstract] OR "ATB"[Title/Abstract] OR "Teeth derived graft"[Title/Abstract]) AND ("PRF"[Title/Abstract] OR "Platelet rich fibrin"[Title/Abstract]) |
| Scopus | TITLE-ABS-KEY (“Autologous bone graft” OR “ATG” OR “Tooth graft” OR “dentin” OR “human dentin” OR “DDM” OR “dentin matrix” OR “dentin graft” OR “ATB” OR “Teeth derived graft”) AND (“PRF” OR “Platelet rich fibrin”) |
| Embase | ('autologous bone graft':ti,ab,kw OR 'atg':ti,ab,kw OR 'tooth graft':ti,ab,kw OR 'dentin':ti,ab,kw OR 'human dentin':ti,ab,kw OR 'ddm':ti,ab,kw OR 'dentin matrix':ti,ab,kw OR 'dentin graft':ti,ab,kw OR 'atb':ti,ab,kw OR 'teeth derived graft':ti,ab,kw) AND ('prf':ti,ab,kw OR 'platelet rich fibrin':ti,ab,kw) |
| Google scholar | intitle: (“Autologous bone graft” OR “ATG” OR “Tooth graft” OR “dentin” OR “human dentin” OR “DDM” OR “dentin matrix” OR “dentin graft” OR “ATB” OR “Teeth derived graft”) AND  intitle: (“PRF” OR “Platelet rich fibrin”) |
| ProQuest | abstract((“Autologous bone graft” OR “ATG” OR “Tooth graft” OR  “dentin” OR “human dentin” OR “DDM” OR “dentin matrix”  OR “dentin graft” OR “ATB” OR “Teeth derived graft”)) AND abstract((“PRF” OR “Platelet rich fibrin”)) |

**Table S1: Specific search strategy for each database**
