# Supplementary material for: Tooth Graft and Platelet‐Rich Fibrin Mixture for Oral Bone Reconstruction and Preservation: A Scoping Review
Source: Clin Exp Dent Res. 2025 Jul 31;11(4):e70160. doi: 10.1002/cre2.70160 (PMC12311611; doi:10.1002/cre2.70160)
Supplement: Supplementary file 3 — Table S3. [file CRE2-11-e70160-s003.docx]

| Author (Year) | Clear demographic characteristics (D1) | Clear patient’s history (D2) | Clear clinical condition of the patient (D3) | Clear diagnostic tests or assessment methods (D4) | Clear intervention(s) or treatment procedure(s) (D5) | Clear post-intervention clinical condition (D6) | Identify adverse events (harms) or unanticipated events (D7) | Takeaway lessons (D8) |
| --- | --- | --- | --- | --- | --- | --- | --- | --- |
| Adamska P  (2024) | Yes | Yes | Yes | Yes | Yes | Yes | Yes | No |
| Vares Y (2022) | Yes | Yes | Yes | Yes | Yes | Yes | Yes | No |
| Khunger A  (2021) | Yes | Yes | Yes | Yes | Yes | Yes | Yes | No |
| Singh A  (2023) | Yes | Yes | Yes | Yes | Yes | Yes | Yes | No |

**Table S3: Risk of bias evaluation for case report studies.**
