# Supplementary material for: Tooth Graft and Platelet‐Rich Fibrin Mixture for Oral Bone Reconstruction and Preservation: A Scoping Review
Source: Clin Exp Dent Res. 2025 Jul 31;11(4):e70160. doi: 10.1002/cre2.70160 (PMC12311611; doi:10.1002/cre2.70160)
Supplement: Supplementary file 4 — Table S4. [file CRE2-11-e70160-s005.docx]

| Author (Year) | Clear inclusion criteria (D1) | Standard and reliable measurement (D2) | Valid methods used for identification of the condition (D3) | Consecutive inclusion (D4) | Complete inclusion of participants (D5) | Clear participants’ demographics (D6) | clear reporting of clinical information (D7) | Clearly reported outcomes (D8) | Appropriate statistical analysis (D9) |
| --- | --- | --- | --- | --- | --- | --- | --- | --- | --- |
| Alrmali A  (2023) | Yes | Yes | Yes | Yes | Yes | Yes | Yes | Yes | Yes |
| Pohl S (2020) | Yes | Yes | Yes | Yes | Yes | Yes | Yes | Yes | Unclear |
| van Orten A (2022) | Yes | Yes | Yes | Yes | Yes | Yes | Yes | Yes | Unclear |
| Andrade C (2019) | Yes | Yes | Yes | Yes | Yes | Yes | Yes | Yes | Yes |
| Melek (2017) | Yes | Yes | Yes | No | No | Yes | Yes | Yes | Unclear |

**Table S4: Risk of bias evaluation for case series studies.**
