# Supplementary material for: Tooth Graft and Platelet‐Rich Fibrin Mixture for Oral Bone Reconstruction and Preservation: A Scoping Review
Source: Clin Exp Dent Res. 2025 Jul 31;11(4):e70160. doi: 10.1002/cre2.70160 (PMC12311611; doi:10.1002/cre2.70160)
Supplement: Supplementary file 5 — Table S5. [file CRE2-11-e70160-s001.docx]

| Author (Years) | True randomization (D1) | Concealed allocation (D2) | treatment groups similar at the baseline (D3) | Blinded participants (D4) | Blinded delivery (D5) | Treatment groups treated identically (D6) | Blinded outcome assessors (D7) | Outcomes measured in the same way (D8) | Reliable outcomes (D9) | Completed follow-up (D10) | Randomized analysis (D11) | Appropriate statistical analysis (D12) | Deviations from the standard RCT design |
| --- | --- | --- | --- | --- | --- | --- | --- | --- | --- | --- | --- | --- | --- |
| Amer (2024) | Yes | Yes | Yes | Yes | Yes | Yes | Unclear | Yes | Yes | Yes | Yes | Yes | NA |
| Gowda (2023) | Yes | Yes | Yes | Yes | Unclear | Yes | Unclear | Yes | Yes | Yes | Unclear | Unclear | NA |
| Abdelraheim (2023) | Yes | Unclear | Yes | Unclear | Unclear | Yes | Unclear | Yes | Yes | Yes | Unclear | Yes | NA |
| Sah (2022) | Yes | Unclear | Yes | Unclear | Unclear | Yes | Unclear | Yes | Yes | Yes | Yes | Yes | NA |
| Serroni  (2022) | Yes | Yes | Yes | Yes | Yes | Yes | Yes | Yes | Yes | Yes | Yes | Yes | NA |
| Mohammed (2021) | Yes | Unclear | Yes | Unclear | Unclear | Yes | Unclear | Yes | Yes | Yes | Unclear | Unclear | NA |
| ElAmrousy  (2022) | Yes | Yes | Yes | Yes | Unclear | Yes | Unclear | Yes | Yes | Yes | Unclear | Yes | NA |
| Ouyyamwongs (2019) | Yes | Unclear | Yes | Unclear | Unclear | Yes | Unclear | Yes | Yes | Yes | Unclear | Yes | NA |

**Table S5: Risk of bias evaluation for randomized clinical trial studies.**
